# Functionalized nanoparticles to deliver nucleic acids to the brain for the treatment of Alzheimer’s disease

**DOI:** 10.3389/fphar.2024.1405423

**Published:** 2024-05-24

**Authors:** Chinenye Edith Muolokwu, Bivek Chaulagain, Avinash Gothwal, Arun Kumar Mahanta, Benjamin Tagoe, Babita Lamsal, Jagdish Singh

**Affiliations:** Department of Pharmaceutical Sciences, School of Pharmacy, College of Health and Human Sciences, North Dakota State University, Fargo, ND, United States

**Keywords:** Alzheimer’s disease, functionalized nanoparticles, gene delivery, nucleic acids, blood-brain barrier, cell-penetrating peptides, targeting ligands

## Abstract

Brain-targeted gene delivery across the blood-brain barrier (BBB) is a significant challenge in the 21st century for the healthcare sector, particularly in developing an effective treatment strategy against Alzheimer’s disease (AD). The Internal architecture of the brain capillary endothelium restricts bio-actives entry into the brain. Additionally, therapy with nucleic acids faces challenges like vulnerability to degradation by nucleases and potential immune responses. Functionalized nanocarrier-based gene delivery approaches have resulted in safe and effective platforms. These nanoparticles (NPs) have demonstrated efficacy in protecting nucleic acids from degradation, enhancing transport across the BBB, increasing bioavailability, prolonging circulation time, and regulating gene expression of key proteins involved in AD pathology. We provided a detailed review of several nanocarriers and targeting ligands such as cell-penetrating peptides (CPPs), endogenous proteins, and antibodies. The utilization of functionalized NPs extends beyond a singular system, serving as a versatile platform for customization in related neurodegenerative diseases. Only a few numbers of bioactive regimens can go through the BBB. Thus, exploring functionalized NPs for brain-targeted gene delivery is of utmost necessity. Currently, genes are considered high therapeutic potential molecules for altering any disease-causing gene. Through surface modification, nanoparticulate systems can be tailored to address various diseases by replacing the target-specific molecule on their surface. This review article presents several nanoparticulate delivery systems, such as lipid NPs, polymeric micelles, exosomes, and polymeric NPs, for nucleic acids delivery to the brain and the functionalization strategies explored in AD research.

## 1 Introduction

Alzheimer’s Disease (AD) is recognized as the leading cause of dementia among people aged 65 and older. It is characterized by the loss of neuronal cells due to the buildup of extracellular amyloid plaques and intraneuronal neurofibrillary tangles in the brain (Bloom, 2014; Singh et al., 2022). This sequence of events eventually leads to reduced cognition, memory loss, and trouble with performing everyday tasks (Burns and Iliffe, 2009). Consequently, it poses a significant burden on global health, affecting millions of individuals and their families. The World Health Organization (WHO) projects an increase in dementia cases globally from about 55 million in 2019 to over 139 million by the year 2050, with AD accounting for 60%–70% of cases (WHO, 2023).^1^ As the population continues to age, the prevalence of AD will be on the rise, making it a major public health concern.

### 1.1 Treatment options for AD

Currently, treatment strategies for AD mainly focus on addressing symptoms rather than treating the underlying cause. Until recently, the Food and Drug Administration (FDA) has approved various treatments, encompassing cholinesterase inhibitors such as donepezil, rivastigmine, and galantamine, along with glutamate regulators like memantine and anti-amyloid monoclonal antibodies such as lecanemab and aducanumab.

#### 1.1.1 Anti-Amyloid Monoclonal Antibodies

Aducanumab, marketed as Aduhelm, marks the first disease-modifying medication approved for individuals with AD. Aducanumab received FDA approval in June 2021 (Dunn et al., 2021). Nonetheless, the European Medicine Agency (EMA) refused marketing authorization for aducanumab in December 2021 because results from clinical studies did not convincingly show the effectiveness of the drug in AD treatment, and brain scan images showed brain swelling or bleeding, questioning the safety of the drug (Villain et al., 2022). As an IgG1 monoclonal antibody, it targets extracellular β-amyloid (Aβ) plaques in the brain, binding to and assisting in removing them. Aducanumab is administered intravenously for an hour every 4 weeks (Dunn et al., 2021; Walsh et al., 2021). Despite its conditional approval, clinical studies on Aducanumab show a decline in Aβ plaque burden, but this reduction does not correlate with any beneficial effects on cognition among patients. The aducanumab FDA approval notice has lifted the spirits of patients living with AD and advocacy organizations. Apart from being the initial treatment aimed at modifying the disease’s pathology, it is anticipated to pave the way for developing comparable therapies in the near future. However, despite receiving accelerated approval from FDA for AD treatment in 2021, Aducanumab is scheduled to be discontinued by its manufacturer, Biogen, in 2024. This decision is part of a strategic reallocation of resources towards other treatments, including Lecanemab, to further advance the development of novel therapeutic approaches. Notably, the discontinuation is not predicated on concerns about safety or efficacy (Biogen, 2024).^2^ The company announced that individuals who are currently participating in clinical trials for Aducanumab will be able to continue doing so until 24 May 2024. In addition, those getting prescriptions will have continued availability until 1 November 2024 (Alzheimer’s Association, 2024).^3^

In January 2023, the FDA approved Lecanemab (Leqembi), a novel disease-modifying human monoclonal antibody targeting amyloid-beta (Aβ), for the treatment of AD. Lecanemab is given intravenously at a 10 mg/kg dose every 2 weeks (Swanson et al., 2021).

Lecanemab demonstrated effectiveness in reducing beta-amyloid accumulation, thereby slowing cognitive decline. It has also exhibited favorable tolerability (Chowdhury and Chowdhury, 2023). As of March 2024, Lacanemab is still under review by EMA (Eisai, 2024).^4^

#### 1.1.2 Cholinesterase inhibitors

At present, cholinesterase inhibitors stand as the primary pharmacological intervention for AD. Clinically, the three principal cholinesterase inhibitors employed are donepezil, rivastigmine, and galantamine. AD is characterized by a concurrent decline in cholinergic neurons and a reduced acetylcholine levels within the brain cortex. Numerous studies have demonstrated that augmenting acetylcholine levels in individuals with dementia contributes to the reduction of cognitive decline. The mechanism of action of cholinesterase inhibitors involves hindering the degradation of acetylcholine, leading to enhanced cholinergic activity, thereby benefiting the patient (Hampel et al., 2019). The FDA approved donepezil in 1996. Its associated side effects include sleep disturbances, irregular heart rhythms, seizures, and nausea (Shintani and Uchida, 1997). Subsequently, in 1997 and 2001, FDA approved rivastigmine and galantamine, respectively, for AD pharmacotherapy. Donepezil and galantamine received approval through the European mutual recognition procedures in 1997 (2011 in the Netherlands) and 2000 respectively, while rivastigmine was approved through EMA in 1998 (Dekker et al., 2019). Similar to donepezil, both rivastigmine and galantamine operate by reversibly inhibiting acetylcholinesterase (AChE), thereby enhancing the intrinsic activity of acetylcholine on cholinergic receptors (McShane et al., 2019). Donepezil is given as an oral tablet at a dose of 5 or 10 mg/day, but for moderate to severe cases of AD, a dose of 23 mg/day oral tablet is administered. Rivastigmine is administered as a transdermal patch at a dose of 13.3 mg every 24 h. Galantamine is used for mild to moderate AD cases, and it is administered orally at 16–24 mg/day (Deardorff et al., 2015). Although cholinesterase inhibitors have been utilized in treating AD, they specifically target symptom relief in patients and do not hinder the progression of the condition. The use of donepezil results in slight improvements in cognitive function but does not improve the treatment costs, caregiver duties, or length of hospital stays (Knowles, 2006).

#### 1.1.3 Glutamatergic modulators

The brain relies on glutamate as its primary neurotransmitter for excitatory signaling. Overstimulation, especially in N-methyl-D-aspartate (NMDA) receptors, can lead to neurodegeneration. However, completely blocking NMDA receptors has led to notable adverse effects. Therefore, the development of memantine, an uncompetitive NMDA receptor antagonist, provides therapeutic advantages by regulating NMDA receptor activation. This strategy aims to safeguard patients from inhibitory effects resulting from excessive activation (Johnson and Kotermanski, 2006). Memantine is employed to manage moderate-to-severe AD in various regions, including the United States, China, Canada, and Europe. It obtained approval from FDA in 2003 and EMA in 2002 (Dekker et al., 2019). In the central nervous system, memantine functions as an antagonist to N-methyl-D-aspartate glutamate receptors (NMDAR), offering a therapeutic approach for AD (Popik et al., 2003). It is administered orally at 5–20 mg/day (Reisberg et al., 2003).

Despite memantine being used to treat AD, its therapeutic benefits mainly target symptom management in patients rather than halting the advancement of the condition. Studies suggest that memantine is proven to be a safe and effective means of improving cognitive function in individuals with advanced AD. However, there is insufficient evidence documenting clinical advantages for those in the early stages of AD (Tampi and van Dyck, 2007).

## 2 Nucleic acids as a therapeutic target for AD

Addressing the genetic foundations of numerous diseases is swiftly transitioning from a theoretical concept to reality. This is exemplified by the recent endorsements from FDA and EMA for several nucleic-acid-based therapeutics (Kulkarni et al., 2021), and this can be attributed primarily to the distinctive benefits offered by nucleic acid drugs. Unlike traditional pharmaceuticals, which predominantly focus on proteins, genetic medications regulate gene expression to elicit therapeutic outcomes. Introducing exogenous nucleic acids into cells to address malfunctioning genes presents an appealing approach for achieving highly targeted, long-lasting, and potentially curative therapeutic outcomes in inherited and acquired disorders. MicroRNAs (miRNAs), small interfering RNAs (siRNAs), and antisense oligonucleotides (ASOs) represent conventional nucleic acid drugs designed to inhibit target genes through complementary binding to the target RNA.

In contrast, plasmid DNAs (pDNAs) and messenger RNAs (mRNAs) are frequently employed to enhance the expression of specific genes. In addition, clustered regularly interspaced short palindromic repeats (CRISPR)/Cas systems exhibit greater versatility, enabling modulation of target gene expression by amplifying, suppressing, and correcting it (Lu et al., 2023). The approval of the nucleic acid aptamer Macugen by FDA and EMA for the treatment of age-related macular degeneration highlights the potential of nucleic-acid based technology for neurological diseases, including AD (Cunningham et al., 2005). The active substance of Macugen, pegaptanib, was designed to block the action of VEGF, a peptide associated with the clinical manifestation of AD. However, Macugen was discontinued by its manufacturer, Eyetech, mainly due to the superiority of other VEGF inhibitors on the market (Louie, 2022). This progress underscores the promise of leveraging nucleic acid-based approaches in developing innovative treatments for AD. Nonetheless, utilizing nucleic acids as therapeutic agents poses challenges due to their vulnerability to nuclease degradation, potential to trigger immune responses, and unfavorable physicochemical properties that hinder their easy entry into cells. The heightened negative charge density of the nucleic acids further impedes cellular uptake by target cells. In contrast to conventional drugs, the distinct characteristics of nucleic acids for therapeutic purposes necessitate delivery through vectors. Overall, the effectiveness of nucleic acid therapy heavily depends on the delivery vectors guiding the nucleic acid to the intended target sites (Lin and Qi, 2023).

### 2.1 Nucleic acids and their mechanism of action

**FIGURE 1:**
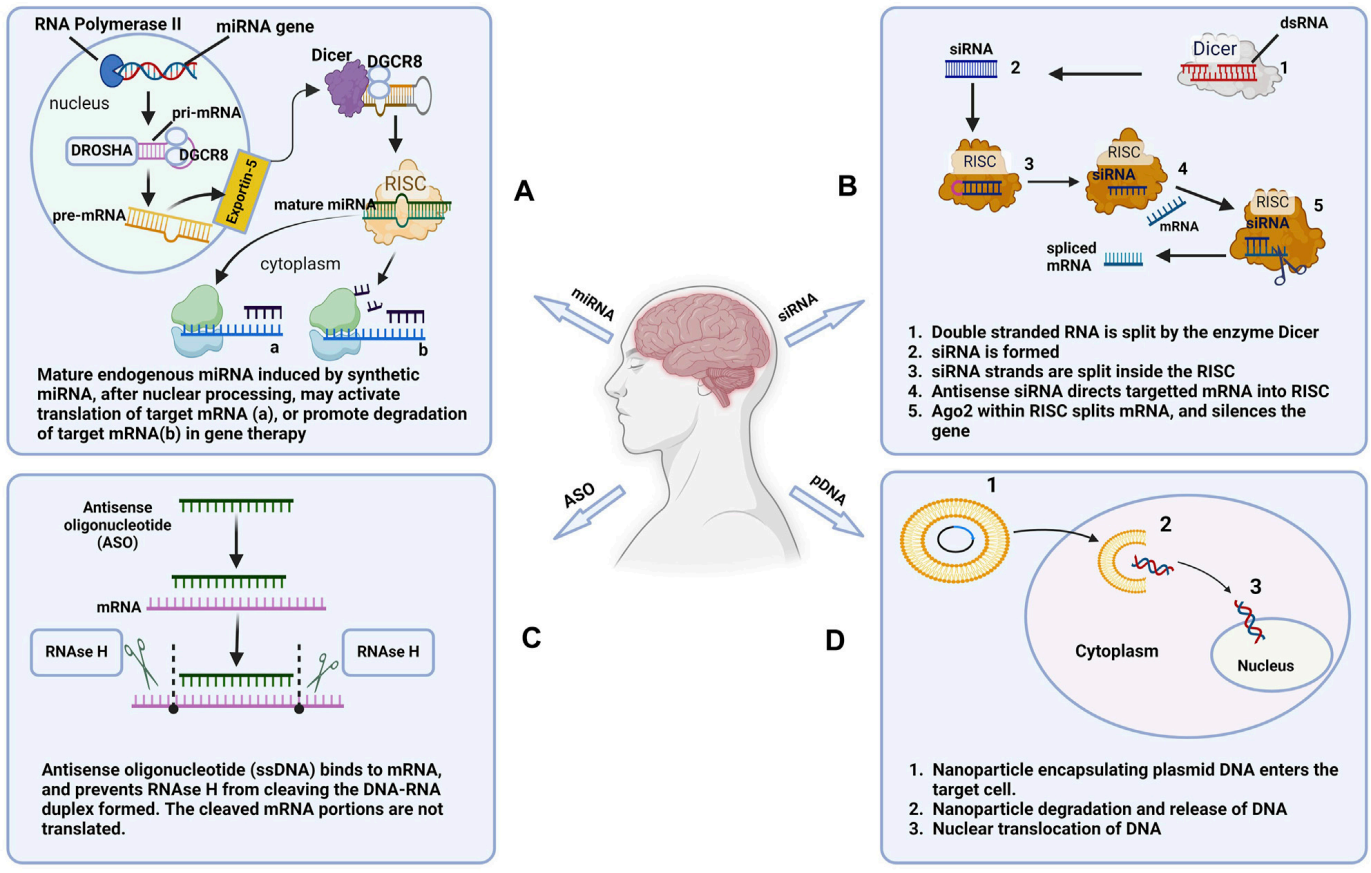
Mechanisms of action of commonly used nucleic acids. **(A)** microRNA (miRNA), **(B)** Small interfering RNA (siRNA), **(C)** Antisense oligonucleotide (ASO), and **(D)** Plasmid DNA (pDNA) used in AD therapy (Created in Biorender.com).

Nucleic acids are derived from monomers called nucleotides. Each nucleotide consists of a five-carbon sugar, a nitrogenous base, and at least one phosphate group. Naturally occurring nucleic acids such as ribonucleic acid (RNA) and deoxyribonucleic acid (DNA), play key roles in gene regulation and transmission of genetic information (Minchin and Lodge, 2019). These endogenous genes encode proteins by specifying the amino acid sequence required for protein synthesis. Factors such as gene mutations and chromosomal damage could lead to the development of genetic disorders (Kapeli et al., 2017). In clinical settings, exogenous nucleic acids have been used to treat genetic disorders by supplementing deficient proteins or inhibiting the expression of malfunctioning genes. In this section, we review the mechanisms of action of nucleic acids-based molecules while highlighting their therapeutic potential in AD therapy (Figure 1).

#### 2.1.1 Plasmid DNA (pDNA)

pDNA usually consists of circular DNA, ranging from 2,000 to 20,000 base pairs. Gene therapy with pDNA involves cloning therapeutically functioning target genes into pDNA (Han et al., 2009). pDNA is frequently derived from recombinant *Escherichia coli* and utilized after purification to remove endotoxins and RNA. Plasmids are efficiently used in gene therapy due to their stability. Following the cell’s absorption of DNA, it must travel to the nucleus. Under certain conditions, pDNA is integrated into the genetic material of the host chromosome and transmitted to the next-generation through chromosome replication and cell division. The genetic information encoded by the pDNA is translated into a protein (Prather et al., 2003). The basic components of pDNA are the elements necessary for maintenance and reproduction in bacteria, as well as the elements necessary for expression in mammals. The components within bacteria encompass replication origins, genes conferring antibiotic resistance, and additional markers for plasmid amplification. Mammalian expression elements comprise of enhancer/promoter sequences derived from mammals or viruses to drive gene expression. These elements typically include the 5′ UTR, containing reporter transgenes, polyadenylation sequences, and introns (Gill et al., 2009). Naturally occurring apolipoprotein E (APOE) gene plays a role in AD by mediating processes such as cholesterol transport, neuronal signaling, amyloid-beta clearance, and synaptic plasticity. As a result, targeting malfunctioning APOE genes in AD patients with APOE pDNA could serve as a viable therapeutic option (Hunsberger et al., 2019).

Currently, novel therapeutic approaches using pDNA are being explored for neurological conditions, including AD. Researchers have explored stimulating neurotrophins such as Brain-derived neurotrophic factor (BDNF) and Vascular endothelial growth factor (VEGF) in the brain. The neurotrophin BDNF induces the expression of Neurosecretory protein VGF (non-acronymic), which has been shown to decline in AD patients. A study has demonstrated that BDNF and VGF play a significant role in cognitive function. Thus, plasmid forms of BDNF and VGF can potentially alleviate AD symptoms (Lin et al., 2015). In a study conducted in animal models of AD, immunization with a plasmid encoding an Aβ1-42 trimeric fusion protein together with Gal4 responsive promoter elements increased the expression of antigens and the generation of anti-Aβ42 antibodies. The results of the study highlight the potential of pDNA in AD therapy (Qu et al., 2010).

#### 2.1.2 Messenger RNA (mRNA)

mRNA is a lengthy, unbranched polynucleotide consisting of a single strand, ranging from 500 to 5,000 nucleotides. The structure consists of a 3′ poly(A) tail 5′ cap, a 3′ untranslated region, a 5′untranslated region, and an open reading frame (ORF). Upon entering cells, the open reading frame section expresses the encoded protein. At the same time, the remaining regions of the mRNA safeguard it and control its translation (Cobb, 2015). Generally, mRNA translation does not require entry into the cell nucleus, as the process can occur inside the cytoplasm.

Furthermore, mRNA does not integrate into the host’s DNA, ensuring efficient expression of proteins in slowly dividing cells, like neuronal cells. In addition, the risk of serious adverse effects such as carcinogenesis is reduced with mRNA therapy (Sahin et al., 2014). The strength of mRNA in disease management encompasses safety, efficiency, and flexibility. By adjusting regulations and optimizing sequences, a notable enhancement in mRNA stability and translation efficiency can be achieved. Messenger RNA has been shown to be an important therapeutic marker in the diagnosis and treatment of AD. For instance, late stages of AD development are characterized by high levels of beta-site amyloid precursor protein cleaving enzyme 1 (BACE1) mRNA. As such, silencing BACE1 mRNA is a viable therapeutic approach (Holsinger et al., 2002). Attenuated levels of BDNF mRNA were found in Alzheimer patients, further highlighting the potential of mRNA gene therapy in the management of AD (Phillips et al., 1991). The therapeutic potential of mRNAs in AD therapy has been emphasized in research studies using animal models. A study demonstrated that mRNA can be utilized to induce the expression of Neprilysin in the brain of mice. This transmembrane protein can break down individual amyloid beta (Aβ) molecules and small clusters, leading to a decrease in the accumulation of Aβ (Lin et al., 2016).

#### 2.1.3 Small interfering RNA (siRNA)

siRNAs are non-coding RNAs with 20–24 nucleotides that can cause post-transcriptional silencing of the desired gene. The first stage of ribonucleic acid interference entails the processing and cleaving of large double-stranded RNA by the enzyme Dicer into small siRNAs (McNamara et al., 2006). Once formed, the siRNA strands are split up inside the RNA-induced silencing complex (RISC). The more stable 5ʹ end of the siRNA strand is usually added to the active RISC complex. Subsequently, the specific mRNA being targeted is directed towards the RISC by the single-stranded antisense siRNA. After alignment, a protein within the RISC complex, Ago2, catalyzes the cleavage of mRNA (Hammond et al., 2000; Song et al., 2004). While siRNA shows promise in drug development, various barriers hinder its widespread clinical application. Naked siRNA exhibits drawbacks such as poor pharmacokinetic behavior, adverse reactions, and stability concerns.

Regarding stability, siRNA’s phosphodiester bond is susceptible to degradation by ribonucleases and phosphatases. In addition, siRNA is rapidly degraded into fragments by endonucleases and exonucleases following systemic administration, reducing the bioavailability of siRNA at the target sites of action. Although siRNA typically operates effectively when its antisense strand perfectly matches the target mRNA, the RNA-induced silencing complex (RISC) can tolerate some mismatches, which may inadvertently result in unintended gene silencing (Hu et al., 2020). Researchers have investigated siRNA effects on gene silencing, specifically targeting genes associated with AD. Genes encoding Presenilin-1 (PSEN 1), amyloid precursor protein (APP), and APOE have been targeted explicitly with siRNA to induce silencing (Taylor, 2023). A study showed that siRNA can be used to knock down the GSK3β gene, reducing amyloid beta levels in the *in vitro* AD models (Gupta et al., 2022).

#### 2.1.4 Antagomirs (antimiRs) and microRNAs (miRNAs) mimics

miRNAs, short in nucleotide length and highly conserved in nature, alter gene expression by imitating endogenous miRNAs. The inhibition of gene expression by miRNA involves the repressing of translation or mRNA degradation. In contrast, antimiRs act by blocking the action of endogenous miRNAs (Jonas and Izaurralde, 2015). The transcription of miRNA by RNA polymerases II or III creates a lengthy primary miRNA with a poly(A) tail and a 5′cap. The microprocessor complex processes primary miRNAs in the nucleus into precursor miRNAs, which are small 70-nucleotide hairpin structures. Exportin 5 facilitates the transportation of precursor miRNAs to the cytoplasm. Consequently, the enzyme Dicer converts them into double-stranded miRNA duplexes of around 22 nucleotides (Ha and Kim, 2014). The therapeutic potential of antimiRs and miRNA mimics has been highlighted in several studies. First, the administration of anti-miR candidate AM-206 significantly increased the brain levels of BDNF. In addition, improvements in neurogenesis and memory were observed in AD animal models treated with AM-206 (Lee et al., 2012). In another study, artificial miRNA targeting acetyl-CoA acyl transferase decreased the accumulation of amyloid beta plaque and improved cognitive performance in an AD mouse model (Murphy et al., 2013). Multiple studies have indicated that distinct miRNAs can control various stages of tau processing, such as splicing and post-translational modifications. The hyperphosphorylation of endogenous tau in the adult forebrain when Dicer is depleted suggests that miRNA plays a direct role in tau-related neuropathies. Subsequently, it was demonstrated that the decrease in the miR-15/107 family in the brains of AD patients is directly associated with this phenomenon. Several miRNAs family have been shown to decrease the expression of mitogen activated protein kinases (MAPK 1/3) (Moncini et al., 2017; Safa et al., 2020). MAPK1/3 phosphorylate tau, in a process linked to the existence of NFTs and senile plaques. A study showed that miR-26a regulated the activity of glycogen synthase kinase 3 beta, which is involved in hyperphosphorylation of tau protein. In addition, glycogen synthase kinase 3 beta is associated with the creation of amyloid beta in the brains of AD patients (Maqbool et al., 2016).

#### 2.1.5 Short hairpin RNA (shRNA)

shRNA molecules can be classified into two primary groups: microRNA-adapted shRNA and simple stem-loop shRNA. The longer microRNA-adapted shRNA design, typically exceeding 250 nucleotides, closely mimics native pri-microRNA molecules found in cells. This design features a shRNA stem structure with microRNA-like mismatches, connected by a loop and bordered by 5′ and 3′endogenous microRNA sequences. This configuration enhances the stability and processing of the shRNA by cellular machinery, leading to more efficient gene silencing through RNA interference (Silva et al., 2005). The simple stem-loop shRNA, 50–70 nucleotides long, is transcribed within the nucleus with the guidance of an RNA Polymerase III promoter. The RNA polymerase II promoter produces a primary transcript with a stem-loop structure resembling a hairpin. In the next step, a complex of RNA-binding domain protein DGCR8 and the RNase III enzyme Drosha processes stem-loop structure inside the cell nucleus (Lee et al., 2003). Exportin 5 facilitates the transportation of the pre-shRNA molecule to the cytoplasm. In the cytoplasm, the pre-shRNA is attached to a complex consisting of the RNase III enzyme Dicer and TRBP/PACT. This compound eliminates the hairpin loop, forming a double-stranded siRNA with 2 nucleotide 3′overhangs. Subsequently, the Dicer complex loads RNA molecules onto the RISC complex’s Ago2 protein and degrades complementing mRNA (Cullen, 2004). ShRNA-induced gene silencing shows potential as a therapeutic strategy for treating AD. Recent studies have shown that Transient Receptor Potential Canonical (TRPC) genes play a crucial role in important cellular processes such as neuronal growth, synapse development, and neuronal differentiation (Tai et al., 2009). Following the injection of a vector carrying TRPC6-specific shRNA into the hippocampal dentate gyrus to knockout TRPC6, there were observed impairments in spatial learning and social recognition memory in the mice that received the treatment after a 4-week duration compared to the control group (Xie et al., 2021).

#### 2.1.6 Antisense oligonucleotides (ASOs)

ASOs are synthetic, short, single-stranded oligonucleotides designed to target mRNA transcripts specifically. They regulate protein production by interfering with the mRNA’s function. Antisense mechanisms encompass various strategies such as recruiting RNase H to cleave mRNA, altering splicing in pre-mRNA, and physically blocking pre-mRNAs. As an illustration, RNase H-mediated cleavage requires the construction of a brief DNA oligonucleotide that forms an RNA-DNA duplex by binding to the target mRNA. Subsequently, endogenous RNase H recognizes this duplex and proceeds to cleave it. Generally, antisense oligonucleotides that regulate pre-mRNA splicing can rectify defective RNA and eradicate splice variants associated with diseases (Bennett and Swayze, 2010).

## 3 Route of administration of nanotherapeutics to the central nervous system (CNS)

**FIGURE 2:**
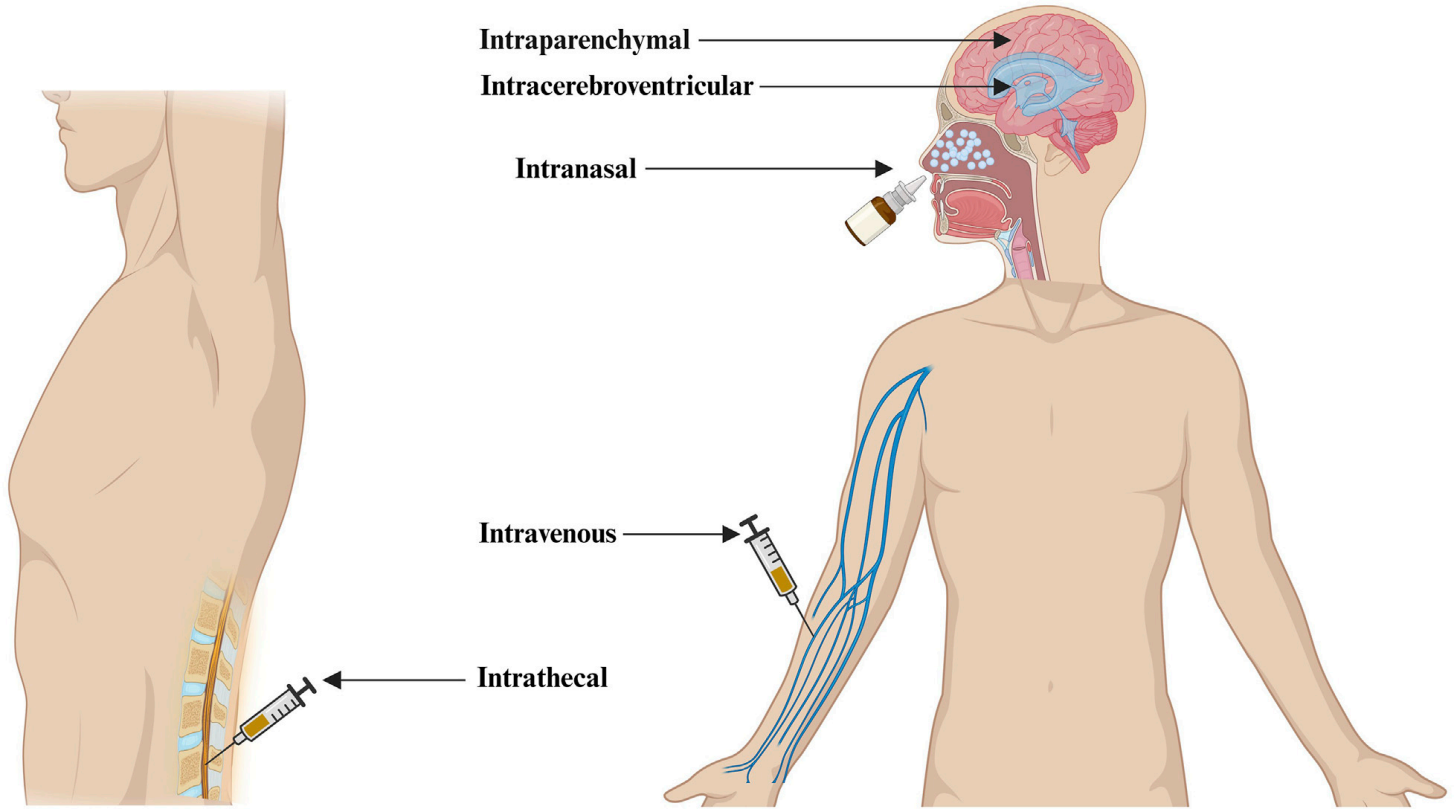
Different routes of administration of nanotherapeutics to the CNS for the treatment of Alzheimer’s disease (Created in Biorender.com).

There are several ways in which nanoparticles (NPs) encapsulating nucleic acids can be delivered to the CNS for the treatment of neurological diseases. Delivery routes such as intravenous, intranasal, intracerebroventricular, intrathecal, and intraparenchymal are the most common routes of administration being explored in research and clinical settings (Figure 2).

Direct CNS delivery using intracerebroventricular, Intrathecal, and Intraparenchymal administration routes has the advantage of delivering a much higher concentration of neurotherapeutics into the cerebrospinal fluid or parenchyma space, improving neurotherapeutics retention in the CNS. The intranasal delivery route holds significant potential for administering nanotherapeutics directly to the brain. Nonetheless, its effectiveness is still being explored due to uncertainties surrounding bioavailability and the challenges of ensuring appropriate dosing (D'Souza et al., 2023). Nanotherapeutics administered through an intravenous route goes directly into the bloodstream and may trigger the activation of complement. This activation can result in the release of anaphylatoxins and proinflammatory mediators. One mechanism is the opsonization of NPs with C3b, which interacts with phagocytes (Moghimi et al., 2019). PEGylation of NPs has emerged as a widely employed strategy to mitigate complement activation. A group of scientists conducted preliminary experiments utilizing PEGylated liposomes, affirming the superiority of carboxy-mediated PEGs over their methoxy-terminated counterparts in suppressing complement activation (Yang et al., 2010). Another study has demonstrated that the incorporation of methoxypoly (ethylene glycol) m-PEG-phospholipid into the liposomal bilayer effectively eliminates the risk of rapid vesicle clearance by mononuclear phagocytic cells, achieved through the suppression of protein adsorption and blood opsonization (Pannuzzo et al., 2020). Despite these polymers’ protective effect on NPs against immune system recognition, available data shows the potential of PEG-specific antibodies to form following intravenous administration of PEG-coated liposomes. As a result, these antibodies lead to quicker PEG-liposome clearance from the bloodstream, altering the pharmacokinetic profile of subsequently administered doses. Hence, it is essential to acknowledge that generating particle-specific antibodies may influence both the safety and efficacy of nanoparticle-based therapeutics (Zolnik et al., 2010). Surface modification of NPs with CPPs and brain-specific targeting ligands have been shown to enhance the intravenous administration of bio-actives to the brain (Arora et al., 2021). We have presented the advantages and limitations of different routes of administration of nanotherapeutics to the CNS (Table 1).

**TABLE 1:** Advantages and limitations of different routes of administration of nanotherapeutics to the CNS.

Route	Advantages	Limitations	References
Intranasal	Therapeutics penetrate the brain directly via the olfactory and trigeminal nerves and can stay longer in the CNS	Factors like mucociliary clearance and enzyme activity in the tract decrease therapeutic efficiency. Additionally, dosing could vary due to inadequate administration. Inter-individual differences in pathologies like congestion and flu may lead to differences in absorption profile	Dong, (2018), Shah et al. (2022), Singh and Shukla (2023)
Intravenous	Direct and fast entry into systemic circulation bypassing first pass effect	Prone to rapid systemic clearance and may pose issues in crossing the BBB	Alam et al. (2010)
Intracerebroventricular	Bypasses the BBB and delivers a high concentration of therapeutics into the CSF, even in small doses	There is a high chance of injury when a needle penetrates the brain tissue, potentially triggering an immune response. Injection into the ventricles could increase intracranial pressure, increasing the chances of CSF leakage, hemorrhage, and CNS infection	Alam et al. (2010), D'Souza et al. (2023)
Intrathecal	Direct administration into cisterna magma of brain. Safer route of administration compared to intracerebroventricular as it does not puncture the brain parenchyma and causes less exposure to enzymatic activity	Delivery via Intrathecal is prone to long transit time to the brain, a high chance of ganglion toxicity, medulla injury, and spinal cord damage	Alam et al. (2010), D'Souza et al. (2023)
Intraparenchymal	Tenfold higher delivery than intracerebroventricular administration and 1,000–10,000-fold better than intravenous administration	Like intracerebroventricular, there is an increased risk of trauma, and needle crossing the brain parenchyma may cause injury. Distribution of therapeutics is localized at the administration site	D'Souza et al. (2023)

## 4 Functionalized NPs for delivering nucleic acids to the brain for the treatment of AD

**FIGURE 3:**
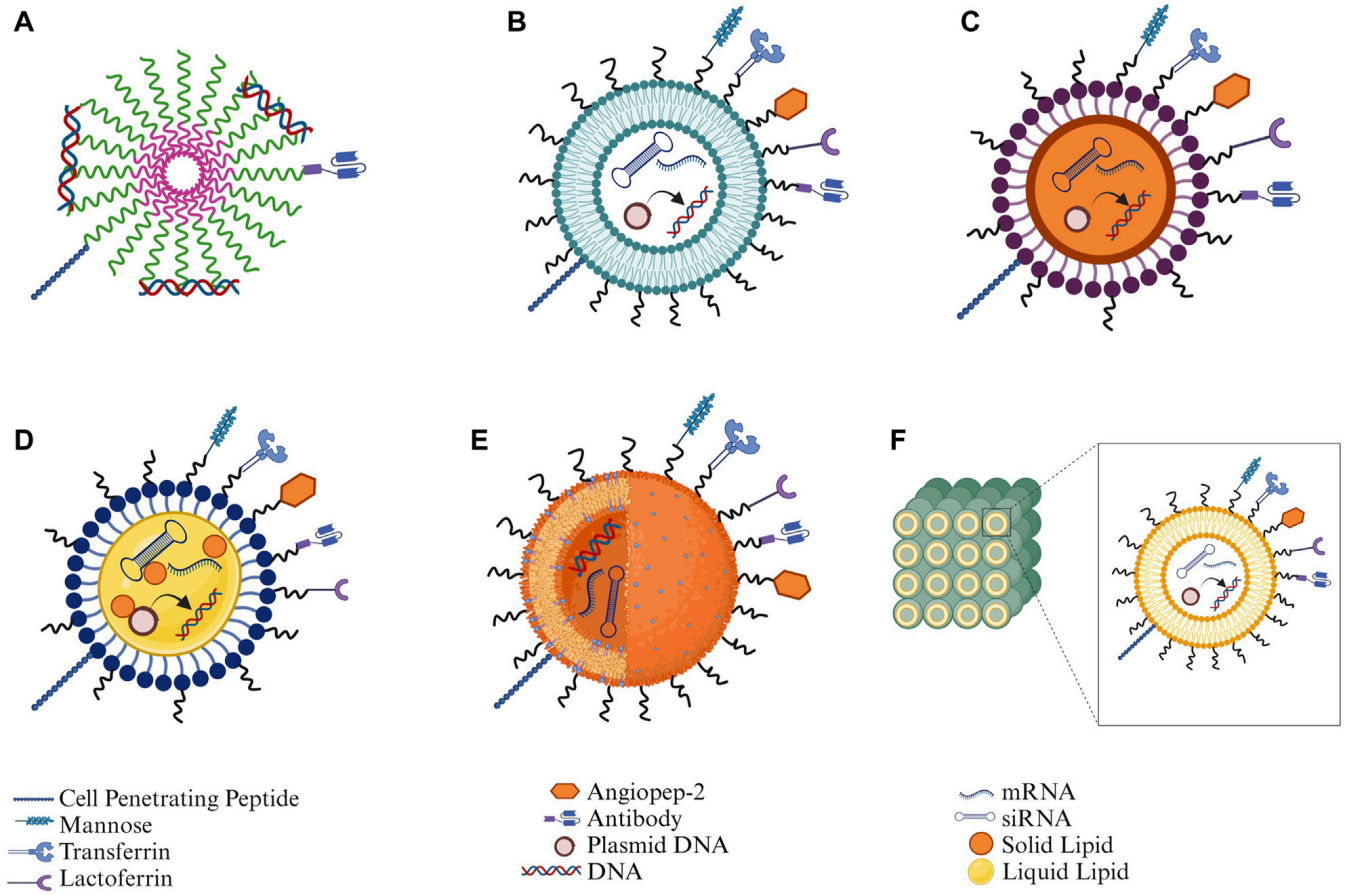
Structural depiction of different types of nanoparticles along with functionalization strategies for nucleic acid delivery: **(A)** Polymeric micelles, **(B)** Liposomes, **(C)** Solid lipid nanoparticles (SLNs), **(D)** Nanostructured lipid carriers (NLCs), **(E)** Polymeric nanoparticles, and **(F)** Cubosomes (Created in Biorender.com).

Functionalized NPs assist in the delivery of nucleic acids to the brain for neurodegenerative disorders, providing a chance to improve transportation across the blood-brain barrier (BBB) and contributing positively to therapeutic payload to specific sites within the brain due to their small size and distinctive features. Recent literature has demonstrated that NPs ranging from 12 to 340 nm in size can cross the BBB. Studies have established that endocytosis, a process influenced by size, allows smaller NPs, particularly those less than 100 nm, to be easily engulfed by cells. Additionally, NPs with a positively charged zeta potential enhance BBB penetration through adsorptive-mediated transcytosis (Lombardo et al., 2020). Functionalization of NPs involves surface modifications utilizing targeting ligands such as CPPs, endogenous proteins, or antibodies. This modification strategy serves to mitigate off-target effects, overcome receptor saturation, and augment internalization via receptor-mediated transport across the BBB. Consequently, this increases the uptake of nanosized particles by neuronal cells, thereby optimizing therapeutic efficacy. Functionalized NPs can be customized to carry various nucleic acid payloads like genes, siRNAs, and ASOs without the concerns of viral replication or immune reactions. Additionally, the ability to scale up production and the flexibility in synthesizing NPs make them more suitable for meeting the requirements of nucleic acid therapies (Figure 3).

### 4.1 Lipid nanoparticles (LNPs)

LNPs are a blend of helper and functional lipids. The main component of functional lipids is an ionizable cationic lipid, and helper lipids such as cholesterol, polyethylene glycol-lipid (PEG-lipid), distearoyl phosphatidylcholine (DSPC), 1,2-distearoyl-sn-glycero-3-phosphorylcholine (DSPC), 1,2-dioleoyl-sn-glycero-3-phosphocholine (DOPC), 1,2-dioleoyl-sn-glycero-3-phosphorylethanolamine (DOPE), 1-stearoyl-2-oleoyl-sn-glycero-3-phosphocholine (SOPC). The ionizable cationic lipids allow for efficient encapsulation of nucleic acids, resulting in a neutral LNP surface charge in circulation, therefore promoting the escape of nucleic acids to cytosol following endocytosis (Kulkarni et al., 2017; Kulkarni et al., 2018). Understanding the many forms of LNPs and their unique properties is essential for the effective delivery of nucleic acid to the brain for the treatment of neurological disorders. Lipid-based carriers can be classified into several categories based on their physicochemical characteristics and how they are being formulated.

#### 4.1.1 Solid Lipid Nanoparticles (SLNs)

SLNs represent lipid-based nanocarrier systems in solid form, primarily comprising lipids such as fatty acids, triglycerides, or modified lipid nanostructures with diameters ranging from 10 to 1,000 nm. These biocompatible vehicles facilitate the distribution of lipophilic and hydrophilic drugs across their solid hydrophobic lipid core (Kaur et al., 2008). Recently, there has been growing interest in SLNs as potential drug delivery systems for brain targeting. SLNs are increasingly recognized for their potential as safe and most cost-effective drug carriers for treating neurodegenerative (Shivananjegowda et al., 2023). Erythropoietin-loaded SLNs showed improved memory in animal models of AD (Dara et al., 2019). *In vitro* and *in vivo* experiments show the multifaceted characteristics of SLNs, such as improved bioavailability of active pharmaceuticals, specificity to target sites, regulated drug release profile, minimal immune response with stealth properties, substantial drug loading capacity, and adaptability to various pharmaceutical as well as biopharmaceutical formulations (Martins et al., 2013). Chitosan-coated SLNs provided total encapsulation of BACE1 siRNA, which aided transportation and delivery to olfactory nerves and protected the nucleic acid against enzymatic degradation, hence preserving its therapeutic effect (Rassu et al., 2017). These distinct properties collectively position SLNs as an ideal and versatile drug delivery system with great potential for advancing nucleic acid delivery to the brain.

#### 4.1.2 Nanostructured lipid carriers (NLCs)

NLCs are colloidal systems characterized by binary lipid mixtures featuring a liquid lipid phase embedded within a solid lipid matrix. Unlike SLNs, NLCs circumvent issues of recrystallization and phase separation of encapsulated pharmacological compounds. They offer greater encapsulation efficiency due to their regulated release and increased stability. Additionally, they minimize drug expulsion during storage, ensuring sustained drug levels in the brain over prolonged periods (Fang et al., 2013; Pardeshi and Belgamwar, 2020; Pinheiro et al., 2020).

#### 4.1.3 Liposomes

Artificial lipid-based bilayered vesicles have become one of the most widely used and effective lipid nanocarriers for neurological disorders. Liposomal nanocarriers offer a significant advantage due to their lipophilic properties, primarily in protecting drugs from degradation, thus enhancing their pharmacokinetic profile (Sonali et al., 2016). A study by a group of scientists proved that the concentrations often employed in animal studies showed no toxicity in the blood while *in vitro* studies showed no interference with the generation of nitric oxide. However, because liposomes lack a targeting molecule, conjugation with peptides, aptamers, or antibodies is crucial to induce targeting effects (Fu et al., 2018; Marcos-Contreras et al., 2020; Ye et al., 2022). Another study showed that functionalized liposomes protect high molecular weight anionic nucleic acids from enzymatic degradation supporting their efficient neuronal targeting and delivery to the brain (Dos Santos Rodrigues et al., 2019b).

#### 4.1.4 Cubosomes

Cubosomes are NPs, which are self-assembling liquid crystalline particles of a certain surfactant with a proper water ratio, as opposed to solid particles. The microstructure of cubosomes gives them unique qualities that are desirable for drug delivery (Garg and Saraf, 2007; Wu et al., 2012). Cubosomes have received much attention recently due to their high drug payload and encapsulation efficiency as well as their ability to stabilize peptides (Landau and Rosenbusch, 1996; Sadhale and Shah, 1999; Barauskas et al., 2005; Azhari et al., 2021), making them probable non-viral vector for nucleic acid delivery to the brain.

#### 4.1.5 Functionalization strategies used in LNPs

LNPs are functionalized via different strategies like surface modification with target ligands such as cell-penetrating peptides, exogenous proteins, and antibodies. CPPs like rabies virus glycoprotein (RVG) peptide, penetratin (PEN) peptide, and exogenous proteins like transferrin (Tf) and lactoferrin (Lf) are some of the common targeting ligands widely used for targeted nucleic acid delivery.

##### 4.1.5.1 Functionalization with cell-penetrating peptides (CPPs)

Researchers have introduced more than 100 CPPs so far which includes cationic and anionic CPPs. CPPs can elevate the cell penetration of a loaded cargo (Sharma et al., 2012). These CPPs have a specific protein sequence known as the protein transduction domain which is responsible for cell penetration (Schmidt et al., 2010). The underlying mechanism of cell penetration of CPPs is still unclear (Reissmann, 2014). The cationic-amphiphilic character of CPPs, such as PEN, helps NPs interact with the lipid components of the cellular membrane and then internalize the cargo into the cell (Zhang et al., 2016). CPPs and brain-specific targeting ligands are being used alone or in combination to facilitate active and efficient gene delivery. A recent study emphasized that formulations incorporating CPPs exhibited brain accumulation levels ranging from 8 to 10 times higher than formulations without the incorporation of CPPs. This observed improvement signifies the efficacy of the K16ApoE CPP in facilitating enhanced delivery of the construct to the brain, emphasizing its potential as a crucial component for optimizing drug transport across biological barriers and elevating therapeutic impact (Ahlschwede et al., 2019).

Transactivating transcriptional activator (TAT), discovered in 1988, is derived from human immunodeficiency virus 1(HIV-1) (Frankel and Pabo, 1988). TAT is considered a powerful transactivator that cells can take up as a viral growth factor. It is composed of 86 amino acids, and since then, it has been used to elevate the transfection potential of LNPs for brain delivery. Transferrin (Tf) is a ligand that harnesses the specificity of Tf-mediated binding to Tf-receptors in brain endothelial cells. An *in vivo* study of liposomes conjugated with TAT and Tf, aiming to improve the penetrability of pharmaceutical formulations through the BBB, shows that TAT-Tf liposomes are efficacious in transporting therapeutic DNA into the brains of mice. This study signifies a promising advancement in targeted drug delivery, highlighting the potential of surface-modified liposomes to facilitate the transport of therapeutic agents across biological barriers for enhanced therapeutic outcomes (Dos Santos Rodrigues et al., 2019a). Nevertheless, TAT presents a potential challenge in AD therapy, as it can trigger Aβ deposition, tau phosphorylation, and subsequent neuronal demise. These considerations are paramount in developing formulations to cross the BBB for AD treatment (Giunta et al., 2009; Di et al., 2016; Hategan et al., 2017). Recent behavioral studies have revealed promising outcomes with a novel fusion peptide combining BDNF with TAT. This fusion peptide effectively targets multiple biochemical pathways in the brain, significantly enhancing spatial memory, restoring memory-associated proteins, and improving cognitive functions in AD-like animal models (Wu et al., 2015).

RVG peptide is a 29-amino acid peptide derived from Rabies virus glycoprotein, a 505 amino acid type 1 membrane glycoprotein, that can bind precisely to neuronal cells’ acetylcholine receptor (nAchR). This allows for nucleic acid entry into neuronal cells via receptor-mediated nucleic acid transport, resulting in efficient gene delivery to the brain (Kumar et al., 2007; Son et al., 2011; Huey et al., 2017; Dos Santos Rodrigues et al., 2019b).

Angiopep-2, a 19 amino acid peptide from the Kunitz domain, binds preferentially to Low-density lipoprotein receptor-related protein-1 (LRP1) and has enhanced penetrability through the BBB (Bertrand et al., 2011; Xin et al., 2012). In a comparative study, a group of scientists reported that SLNs loaded with Angiopep-2 led to prolonged survival of the animal due to increased drug delivery level to the glioma (Kadari et al., 2018). Covalent surface modification of SLN with synthetic decapeptide derived from the receptor binding region of human Apolipoprotein E (mApoE) enhances penetration of NPs when delivered intra-nasally (Dal Magro et al., 2017).

##### 4.1.5.2 Functionalization with exogenous proteins

Tf-liposomal formulation improved the transport of poor brain penetrability agents to the brain in both *in vitro* and *in vivo* studies (Chen et al., 2016). Lactoferrin (Lf) is a cationic iron-binding protein belonging to the transferrin family, characterized by multifaceted functionalities such as anticarcinogenic and anti-inflammatory properties. The Lf exhibits diverse interactions, including engagement with the LDL receptor-related protein, facilitating its transportation to the brain through receptor-mediated endocytosis (Huang et al., 2007). The Lf expression has been observed to be significantly upregulated in both neurons and glia in AD, indicating potential for brain targeting (Kawamata et al., 1993). A study formulated Lf-liposomes, which acts as a brain-specific targeting ligand. The formulation exhibited an improved absorption profile in primary brain capillary endothelial cells, a more remarkable ability to penetrate the BBB *in vitro*, increased accumulation of the formulation in the brain, and decreased cellular toxicity compared to conventionally formulated liposomes (Chen et al., 2010). Surface Lf has also been shown to efficiently transport Nerve Growth Factor (NGF) as Lf/NGF-liposomes across the BBB, inhibiting the degeneration of SK-N-MC cells with Aβ-induced neurotoxicity (Kuo and Wang, 2014).

In another study with plasmid NGF and plasmid BDNF using CPPs and ligands such as Tf and mannose (MAN), the formulation facilitated enhanced cell penetration and effective receptor targeting to deliver nucleic acids to the brain, showing positive therapeutic responses *in vivo* (Rodrigues et al., 2020).

##### 4.1.5.3 Functionalization with antibodies

Recently, it was revealed that a new collection of antibodies targeting receptor-mediated transcytosis (RMT) was shown to bind both the human and mouse BBB in brain tissue sections. To improve brain-specific delivery, a study created an efficient RMT antibody-targeted liposomal system employing antibody (scFv46.1). scFv46.1-modified liposomes were shown to be absorbed by cells *in vitro*. *In vivo* evaluations showed that antibody-liposome loaded with pralidoxime accumulated in the brain ten times higher than the liposomal control group. These results point to the antibody-liposomal formulation’s potential as a tailored synthetic vehicle for CNS medication delivery (Ye et al., 2022).

Furthermore, using Tf as a targeting ligand has limitations, including the potential to saturate Tf-receptors in the body, thereby interfering with iron uptake. To address this issue, antibodies binding to Tf-receptors through non-Tf sites are being explored as an alternative. OX26, an anti-Tf-receptor monoclonal antibody, interacts with the Tf-receptor’s extracellular domain without hindering Tf-binding in rats. Like Tf, OX26 can be used to conjugate drug carriers for BBB penetration. Human basic fibroblast growth factor (bFGF), a potent neuroprotective agent, was conjugated with Streptavidin and OX26, and brain uptake of neurotrophin bFGF was seen to increase (Wu et al., 2002). For neuroimaging in AD, radio-iodinated Aβ (125I-Aβ40) can be linked to OX26 using a streptavidin-biotin linker after intravenous injection (Wong et al., 2019).

#### 4.1.6 Functionalized LNPs for nucleic acid delivery in AD

The transport and transfection of ApoE2 gene in the *in vitro* BBB model were markedly improved by dual functionalized liposomes with CPPs such as RVG, PEN, and the brain targeting ligand Mannose (MAN). Despite the inherent challenges in gene delivery to the brain, dual-functionalized liposomes successfully targeted the brain. They expressed the genetic cargo in brain cells, resulting in considerably elevated ApoE expression in the brains of C57BL/6 mice (Arora et al., 2021). This emphasizes how bi-functionalized liposomes could be an efficient gene delivery method in the treatment of AD. In the brains of three-month-old APP/PS1 mice, liposomes dual modified with PEN and Tf encasing chitosan-pNGF complexes boosted NGF levels, stimulated the formation of new cells, and decreased the levels of harmful soluble and insoluble Aβ peptides (Rodrigues et al., 2020). BDNF gene therapy utilizing bi-functionalized liposomes increased pre- and post-synaptic protein levels above baseline levels in age-matched wild-type animals. Furthermore, compared to untreated age-matched transgenic AD mice controls, a significant decrease in the plaque load was seen in treated age-matched AD groups. These findings imply that amyloid pathology may be prevented or attenuated with BDNF restoration via neurotrophin therapy (Arora et al., 2022).

Additionally, the delivery of BACE1 siRNA through functionalized SLNs containing a short peptide of RVG (RVG-19) and coated with chitosan altered the system’s positive Zeta Potential, mucoadhesiveness, and the ability for the siRNA to penetrate epithelial cells, improving intracellular transport (Rassu et al., 2017). *In vivo* and *in vitro* data of PEGylated anionic liposomes demonstrated more efficient packaging of siRNA, leading to BACE1 gene silencing; roughly a 60% reduction in BACE1 mRNA compared to the untreated group and 30% protein silencing (Tagalakis et al., 2014).

### 4.2 Polymeric micelles

Polymeric micelles are composed of amphiphilic block copolymers which aggregate to form nano scale assemblies (1–200 nm). There are two functional sections in polymeric micelles, the inner part is known as the core, and the outer as the corona. Commonly, the core and corona are composed of hydrophobic and hydrophilic polymers respectively. The corona is responsible for *in vivo* pharmacokinetics whereas the core controls the drug entrapment, drug release, and stability properties. The exclusiveness of polymeric micelles is associated with their structural construction; the inner core can encapsulate the hydrophobic drugs, whereas the corona can be tailored to achieve the specific desired characteristics (Gothwal et al., 2016). As discussed in this review, nucleic acid delivery to the brain is challenging. Therefore, polymeric micelles have been extensively used in brain-targeted nucleic acid deliveries to curtail AD progression.

#### 4.2.1 Functionalization strategies used in polymeric micelles

Polymeric micelles have been functionalized with different CPPs to achieve higher cell penetration. Available literature shows different CPPs such as Tat, RGD, and PEN were used. Recently, these CPPs have been extensively used to improve the cell-penetrating potential of polymeric micelles. For example, study showed that TAT functionalized poly-(N-3-carbobenzyloxy-lysine) (CPCL) polymeric micelles improved the gene transfection (Wang GH. et al., 2018). Similarly, PEG-PCL polymeric micelles functionalized with TAT improved *in vitro* cell penetration (Ming et al., 2019). The TAT CPPs have been explored for intranasal brain delivery of siRNA (Kanazawa et al., 2013). Even multi-CPPs functionalization has improved the transfection potential of polymeric micelles. For instance, RGD/TAT functionalization on poly (ethylene oxide)-block-poly(3-caprolactone)(PEO-b-PCL) polymeric micelles also improved *in vitro* co-delivery of bio-actives (Xiong et al., 2010). In another study, multi functionalized chitosan polymeric micelles showed effective transport across the BBB (Lamptey et al., 2022).

Besides TAT, PEN has been documented as a widely used CPP in elevating nanocarrier internalization across the BBB. It is composed of 16 amino acid sequences (Khafagy El et al., 2010; Gartziandia et al., 2016). The available literature confirms that PEN peptide can enhance the BBB permeability without exerting any cytotoxicity to the tissues (Liu et al., 2014; Nielsen et al., 2014). Multi-functionalized PEN tagged with chitosan polymeric micelles have shown higher *in vitro* transfection without causing any significant toxicity (Layek and Singh, 2013). Higher gene transfection was also achieved in the brain when multi-functionalized chitosan polymeric micelles were tagged with PEN (Gothwal et al., 2023a; Gothwal et al., 2023b). Similarly, Angiopep CPP was explored with PEG-PCL polymeric micelles for effective drug delivery across the BBB (Xin et al., 2011). RVG decorated polymeric micelles demonstrated its promising siRNA delivering potential to the brain (Huo et al., 2015). Limited literature availability on applications of CPP functionalized polymeric micelles in AD shows that this area can be explored for developing effective treatment strategies against AD.

#### 4.2.2 Functionalized polymeric micelles for nucleic acid delivery in AD

Functionalized polymeric micelles have proved their potential in gene delivery against AD. A study investigated the transfection potential of polymeric micelles to deliver siRNA targeting BACE1 and APP (therapeutic targets of AD). Linear polyethyleneimine (LPEI) was grafted with polyethylene glycol (PEG) and loaded with siRNA. The *in vitro* outcomes showed selective knockdown in the mouse neuroblastoma (N2a) cells, and *in vivo* imaging proved the accumulation of polymeric micelles in the brain of mice. Additionally, *in vivo* results demonstrated the efficient knockdown of BACE1 in the brain (Shyam et al., 2015). The CPP functionalization improved gene transfection significantly.

Another group of scientists reported TAT functionalized polyethylene glycol (PEG)-polycaprolactone (PCL) polymeric micelles to deliver siRNA/dextran (10 kDa) to the brain via intranasal administration. The rats were sacrificed after 15 min and 1 h of intranasal and intravenous administration. The *in vivo* research findings provided higher transfer of siRNA to the brain through intranasal administration over intravenous after 1 h of administration (Kanazawa et al., 2013). Similarly, our laboratory has also explored the multi-functionalized chitosan polymeric micelles for comparative analysis of intranasal and intravenous administration. The chitosan polymeric micelles were functionalized with oleic acid (OA) mannose followed by CPPs- TAT and MAN. The multi-functionalized chitosan polymeric micelles were polyplexed with pVGF and investigated for transfection in C57BL/6J mice through intranasal and intravenous administration. Our findings showed higher transfection of pVGF in the multi-functionalized chitosan polymeric micelles in intranasal and intravenous groups over un-functionalized polymeric micelles. Overall higher transfection was observed in the intranasal administered animal groups over intravenous treated groups (Lamptey et al., 2022). We further explored PEN-CPP functionalized chitosan polymeric micelles for intranasal administration of pVGF into the brain. The chitosan was multi-functionalized with oleic acid (OA), followed by PEN and MAN, and finally polyplexed with pVGF. After intranasal administration, the multi-functionalized polymeric micelles showed significantly higher transfection in the mice brains (Gothwal et al., 2023a). In a different study, pApoE2 was used with PEN-conjugated multi-functionalized polymeric micelles and investigated for brain transfection after intranasal administration. The *in vitro* findings showed higher transfection of pApoE2 in primary astrocytes and primary neurons by dual-functionalized micelles. The *in vivo* results were also in the notion of *in vitro* findings, multi-functionalized polymeric micelles-treated mice showed higher ApoE2 expression in the brain over un-functionalized polymeric micelles (Gothwal et al., 2023b).

Polymeric micelle is a promising nanocarrier for gene delivery but limited available literature shows that polymeric micelles can be explored for gene delivery to the brain in AD. Functionalization of polymeric micelles with CPPs can serve as a promising nanocarrier platform in gene delivery to develop effective treatment approaches against AD progression. Polymeric micelles can also be explored with other ligands, including antibodies, amino acid sequences, or in combination to improve the targeted delivery across the BBB. This approach could be effective in targeting different receptors abundantly present on the BBB.

### 4.3 Exosomes

Exosomes are vesicular structures of endo-lysosomal origin ranging from 30 nm to 150 nm in size secreted by cells into extracellular fluids such as blood, cerebrospinal fluid (CSF), urine, saliva, etc. They consist of double-layered lipid membranes interspersed with transmembrane proteins encapsulating nucleic acids, proteins, or bioactive molecules in their aqueous core (Kalluri and LeBleu, 2020). The composition of exosomes is a function of the cell source and its pathological state. The common proteins in exosomes are either of endosomal origin, i.e., annexins, flotillin, tetraspins, or cell surface proteins collagen, integrin, and galectin. The lipid components include cholesterol, ceramides, phosphoglycerides, and short and long chain fatty-acyl side chains. The composition of the exosomes has its effect on physiochemical properties but is not limited to blood opsonization, blood circulation, tissue penetration, or renal clearance (Conlan et al., 2017). Although the functions of exosomes are yet to be established, it is suggested that they are involved in the removal of unnecessary constituents from the cell, like proteins, lipids, or nucleic acids (Kowal et al., 2014) to maintain cellular homeostasis and combat stress (Han et al., 2022), facilitating cellular communication between cells and maintaining the extracellular matrix.

**FIGURE 4:**
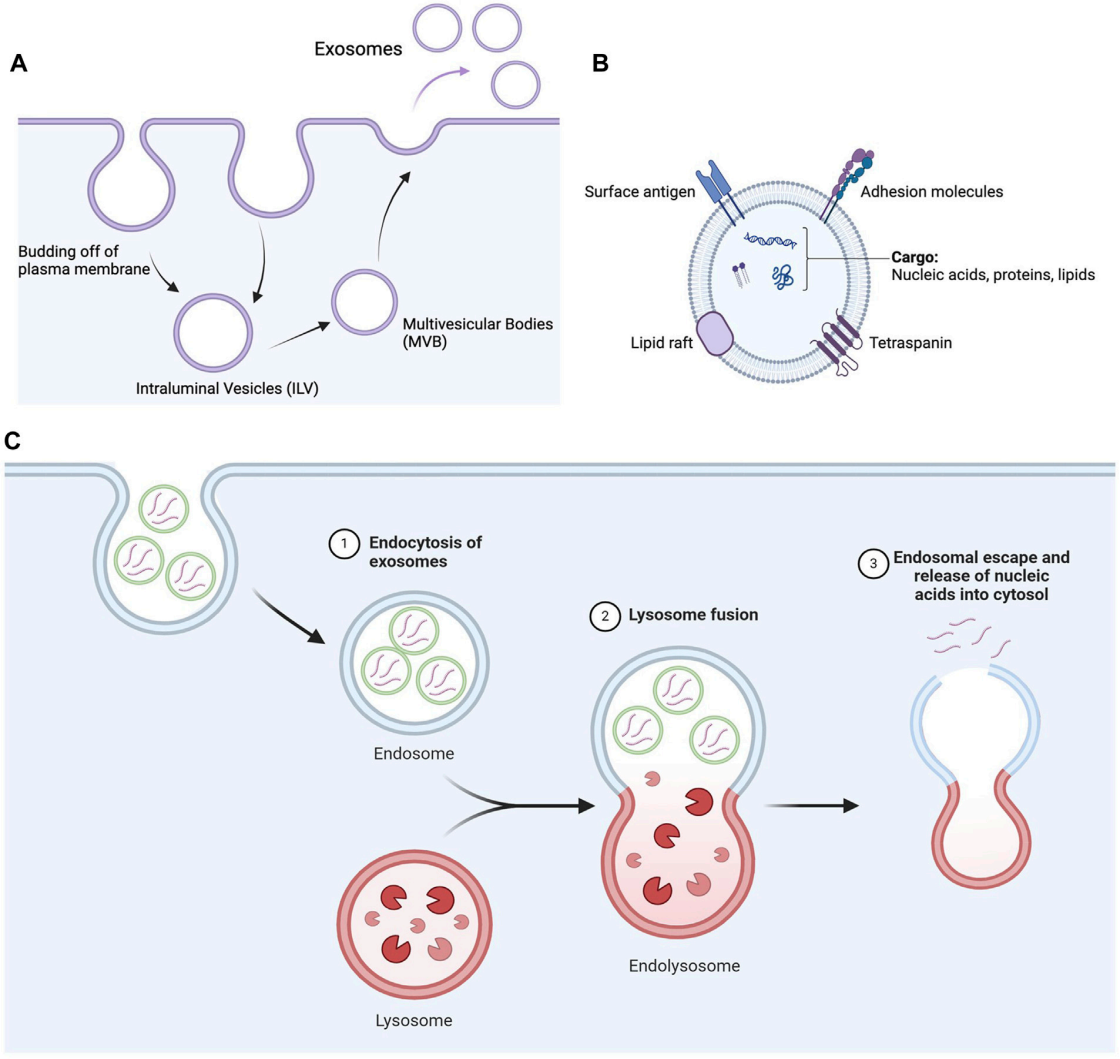
Secretion of exosomes and nucleic acid delivery mediated by exosomes. **(A)** Schematic flow of formation of exosomes inside cells. The invagination of the plasma membrane forms ILV, which matures into MVB. MVB then finally releases exosomes from the plasma membrane. **(B)** Schematic diagram of exosomes representing their lipid architecture, protein markers, and encapsulated contents. **(C)** Exosomes are internalized by cells through endocytosis, such as clathrin-mediated endocytosis and micropinocytosis, depending on the nature of the exosomes and cell type. The internalized exosomes then fuse with lysosomes to form endolysosomes, eventually escaping the endolysosomal pathway to release nucleic acids into the cytosol (Created in Biorender.com).

The formation of exosomes starts with the invagination of the plasma membrane into the cytosol to form an intraluminal vesicle (ILV) (Edgar, 2016). Various proteins of ILVs are recycled back into the plasma membrane, and additional cargos are introduced into them to form multivesicular bodies (MVBs). MVBs either fuse with lysosomes to be degraded or fuse with the plasma membrane to release exosomes into the cytosol (Han et al., 2022) (Figure 4).

The most used method for isolating exosomes includes ultracentrifugation in combination with density gradient centrifugation. This method is labor intensive and time-consuming and requires large amount of samples for isolation. As a result, advanced methods like ultrafiltration or affinity-based capture of exosomes are used that are quick and yield a high amount of exosomes (Zeringer et al., 2015).

#### 4.3.1 Functionalization strategies used in exosomes

Due to their biological origin, exosomes are non-immunogenic, biocompatible, and physiologically stable (Salunkhe et al., 2020). As a result, they are getting increased attention from the drug delivery community towards delivering cargo ranging from small molecules, nucleic acids, and proteins for therapeutic and diagnostic purposes (Donoso-Quezada et al., 2020). The tropism of exosomes is governed by the cell type during isolation, the membrane composition, and the pathological state of the host. Biodistribution studies in mice reported that administration of these exosomes *in vivo* results in their distribution mostly in the liver, spleen, kidney, lung, and gastrointestinal tract while being cleared rapidly from the blood (Choi et al., 2021). Localization towards the brain was minimal (Tian et al., 2018). Engineering exosomes, thus, is an innovative approach to enhance the utility of exosomes in CNS disorders, including AD. Here are some engineering strategies adopted to functionalize exosomes and enhance their delivery to the brain (Table 2).

**TABLE 2:** Functionalization strategies used in exosomes.

Functionalization category	Technique applied	References
Physical	Sonication, extrusion, freeze-thaw	Fuhrmann et al. (2015), Wu et al. (2021)
Chemical	Click chemistry	Tian et al. (2018), Cui et al. (2019), Haroon et al. (2024)
Biological	Chimeric DNA	Kim et al. (2020), Choi et al. (2021), Yu et al. (2021)

##### 4.3.1.1 Chemical functionalization of exosomes

The surface of exosomes is enriched with amine/carboxylic acid-terminated phospholipids and transmembrane proteins, which can be functionalized by targeting moiety through a direct chemical reaction (Tian et al., 2018). Click chemistry is the process of choice for chemical functionalization strategy compared to conventional chemical reactions, which involve noxious solvents and reaction conditions that are detrimental to the integrity of exosomes (Salunkhe et al., 2020). Click chemistry reactions are quick, executed in aqueous solvent conditions, generating high yield, and have less rigorous separation procedures. Some click chemistry reactions employed in exosomes are thiol-maleimide coupling, EDC/NHS coupling, and azide-alkyne cycloaddition coupling (Rayamajhi and Aryal, 2020).

##### 4.3.1.2 Physical functionalization of exosomes

The fluid nature of the exosome membrane has the property to reassemble into its original vesicular organization after brief disruption by physical forces. This property is the core principle of the functionalization achieved through physical forces. The physical forces used to dissemble the membrane integrity are sonication, extrusion, and freeze-thaw cycles (Haney et al., 2015). Additionally, a hydrophobic insertion strategy using functionalized liposomes is also used. In this, after the application of physical forces, the disrupted exosomes are exposed to functionalized liposomes, which ultimately results in a hybridized liposome-exosome system with targeting ligands (Rayamajhi and Aryal, 2020). One fundamental flaw with this method is the release of internal constituents during the process.

##### 4.3.1.3 Biological functionalization of exosomes

The protein machinery of exosome-producing cells can be exploited to engineer transmembrane protein fused with targeting ligands as a biological functionalization method. Lamp, GPI, CD63, CD9, CD81, etc., are the transmembrane protein cohort in exosomes that can be used as a functionalization base (Rayamajhi and Aryal, 2020). Essentially, exosome-producing cells are loaded with expression vectors (plasmids/viruses) containing chimeric genes where the targeting ligand genetic code is fused with the transmembrane protein genetic code. The exosome-producing cells process these chimeric genes to produce transmembrane protein expressing targeting ligand on its surface.

#### 4.3.2 Functionalized exosomes for nucleic acid delivery in AD

A group of scientists tested biologically functionalized exosomes to downregulate BACE1 protein via siRNA for the management of AD. The researchers isolated exosomes from the culture of primary dendritic cells of C57Bl/6 mice bone marrow. The biological functionalization was achieved through plasmid constructs encoding CNS-specific peptide RVG on the genetic backbone of transmembrane exosome protein Lamp2b. siRNA was loaded by electroporation into the exosomes and injected intravenously. The functionalized exosomes demonstrated significant knockdown of BACE1 protein (66% ± 15%, *p* < 0.001) and β amyloid 1-42 (55%, *p* < 0.05), a critical pathological marker of AD, suggesting its application in the management of AD (Alvarez-Erviti et al., 2011). A recent study investigated the therapeutic efficacy of nucleic acid microRNA (miRNA) exosomes against AD. MiR-29a and miR-29b, the family of miR-29 family, are downregulated in AD and play a critical role in regulating BACE1 and neuronal apoptosis. As a result, the researchers used exosomes isolated from bone marrow mesenchymal stem cells (r-BMSCs) for miRNA delivery. The exosomes were biologically modified with vectors consisting of miRNA constructs such that released exosomes were rich in miR-29. The isolated exosomes were then injected intrahippocampally into the Wistar rat model of AD. A significant increase in miR-29 expression was observed in the hippocampus of the rats (*p* < 0.05), which resulted in a reversal of cognitive impairment observed in the Barnes maze (Jahangard et al., 2020).

### 4.4 Polymeric NPs

Polymeric NPs are colloidal NPs used as nanocarrier platforms for drugs and nucleic acid deliveries against different ailments (Reis et al., 2006). Polymeric NPs have shown greater advantages over other nanocarriers, i.e., being biodegradable, compatibility with therapeutic agents, improved shelf-life, and improved release kinetics (Reis et al., 2006; Davis, 2009). The application of functionalized polymeric NPs for nucleic acid delivery in CNS disorders is a promising approach in medicine. Herein, we discuss various functionalization strategies of polymeric NPs for transporting therapeutic genes across the BBB for the treatment of AD.

#### 4.4.1 Functionalized polymeric NPs for nucleic acid delivery in AD

Current strategies for nucleic acid delivery across the BBB have some limitations for clinical translation, such as the risk of inflammation and invasive administration techniques (Singer et al., 2005; Shyam et al., 2015). The functionalization of polymeric NPs offers excellent targeting potential for delivering nucleic acid into the brain without triggering any adverse response. Recently, functionalized PEGylated Poly (2-(N,N-dimethylamino)ethylmethacrylate) (PEG-PDMAEMA) NPs were used to deliver siRNA to the amyloid plaque precisely in AD mice. The NPs were functionalized with QSH and CGN. The d-peptides QSH and CGN also play an important role in delivering the siRNA. CGN is a *d*- CGNHPHLAKYNGT, and a phase displayed *l*-peptide that has the affinity to bind with brain capillary endothelial cells whereas, QSH, a *d*-QSHYRHISPAQV specifically binds to Aβ_1−42_ and selectively targets the amyloid plaques. An intravenous administration of the functionalized NPs containing siRNA led to higher accumulation around the Aβ plaques in transgenic AD mice brain and inhibited BACE1 production, thereby decreasing Aβ formation (Zheng et al., 2017).

Similarly, a group of researchers designed PEGylated poly (2-(N,N-dimethylamino) ethyl methacrylate) (PEGPDMAEMA) polymeric NPs for delivering siRNA across the BBB specifically to the neurons. The nanocarrier was modified with CGN and Tet1 targeting ligands. After caudal vein administration in APP/PS1 transgenic mice, a notable decrease in BACE1 mRNA level and Aβ plaques were observed. Additionally, significant hippocampal neurogenesis was also observed (Wang P. et al., 2018).

The delivery of BACE1 siRNA to neuronal cells helps to improve cognitive dysfunction in transgenic AD mice. Glycosylated polymeric siRNA (Gal-NP@siRNA) nanomedicine has been developed to target BACE1 (Zhou et al., 2020). A salt bridge of guanidinium-phosphate (Gu^+^/PO3^4−^) was used to stabilize the Gal-NP@siRNA through hydrogen bonding. The glycosylated nano delivery system enhanced the BBB penetration through glucose transporter-1 receptors. Administration of Gal-NP@siRNA in APP/PS1 transgenic AD mouse model specifically targets BACE1 and suppresses the expression of BACE1, thereby decreasing amyloid-β formation (Zhou et al., 2020).

Another study developed a functionalized polymeric nanoparticle based on PEGylated dendrigraft poly-L-lysines (DGLs) for co-delivering BACE 1-AS shRNA and peptides. The aim was to reduce Aβ plaques and inhibit p-tau associated fibrils for the treatment of AD. The DGLs were modified with brain-targeted RVG29 and D-peptide through a bi-functional PEG linker, resulting in DGLs-PEG-RVG29-D-peptide NPs. Multiple dosing treatments in transgenic AD mice revealed that the therapeutic gene downregulated the expression of BACE1, resulting in the reduction of Aβ plaques. Additionally, the formation of intracellular tau-fibrils decreased due to the inhibition of tau-fibril formation by D-peptides (Liu et al., 2016).

BDNF has been reported as a key element in neurodegenerative diseases. However, this therapy has some drawbacks, such as exogenous protein safety, including other side effects like neuropathic pain and seizures. Recently, poly (β amino esters) polymers (PBAE) NPs-based delivery of BDNF mRNA was reported for AD. Neuron-specific miRNA targeting modification was done on mRNA to prevent BDNF protein expression in neurons. The astrocytes released BDNF to support the neuronal function, significantly increasing memory improvement in the double transgenic AD mice model (APP/PS1) (Li et al., 2023).

The Rho/ROCK pathway is believed to control neuronal loss and inhibition of axonal growth associated with AD. Blocking the Rho/ROCK pathway could be a possible treatment to promote neuritic extension and regenerate the axon. Therefore, ROCK II-siRNA was delivered to the brain using polyethyleneimine (PEI) NPs modified by PEGylation (PEG). Administration of this formulation by intracranial injection in a senescence-accelerated mouse (SAM) model of AD was found to improve significant cognitive impairment by enhancing Choline acetyltransferase (ChAT) activity in the hippocampus region (Wen et al., 2014).

In addition, RVG29-modified PEGylated PLGA NPs were used to deliver BACE1-AS shRNA-encoded plasmid and epigallocatechin-3-gallate (EGCG) to the brain. Therapeutic (shRNA) gene and EGCG delivery in double transgenic (APP/PS1) mice showed the downregulation of BACE1 enzyme as well as amyloid-beta because of synergistic therapeutic effect (Lv et al., 2020) (Table 3).

**TABLE 3:** Summarized preclinical studies semonstrating the efficacy of functionalized NPs used for nucleic acids delivery to the brain for the treatment of AD.

NPs	Functionalization strategy	Nucleic acids	Route of delivery	Outcome	References
Functionalized LNPs	Chitosan-RVG-9R-SLN	BACE1 siRNA	Intranasal	Improved siRNA’s penetration ability into the epithelial cells	Rassu et al. (2017)
	Anionic PEGylated liposomes with Cationic Targeting peptides	BACE1 siRNA	Intravenous	BACE1 gene silenced	Tagalakis et al. (2014)
	Chitosan-CPP-MAN-liposomes	Plasmid ApoE2	Intravenous	Elevated expression levels of ApoE2 in C57BL/6 mice	Arora et al. (2021)
	Chitosan-PenTf liposomes	Plasmid NGF	Intravenous	Raised levels of NGF in the brain, promoted the growth of new cells, decreased the ratios of Aβ1-42 and Aβ1-42/Aβ1-40, and raised levels of synaptic markers	Rodrigues et al. (2020)
	Chitosan-CPP-MAN-Liposomes	Plasmid BDNF	Intravenous	Increased level of synaptic proteins, decreased amyloid beta synthesis, decreased plaque load, and enhanced cell division	Arora et al. (2022)
	Chitosan-CPP-MAN-Liposomes	Plasmid VGF	Intravenous	Enhanced transfection and increased VGF level ∼ 2-fold higher	Arora and Singh (2021)
Polymeric micelles	Poly(ethylene glycol) (MPEG)/polycaprolactone (PCL) copolymers conjugated with CPP, Tat (MPEG–PCL–Tat)	siRNA	Intranasal	Improved brain delivery over intravenous administration	Kanazawa et al. (2013)
			Intravenous		
	Linear polyethyleneimine (LPEI) grafted with polyethylene glycol (PEG)	BACE1 siRNA	Intracerebroventricular	Efficient knockdown of BACE1 in the brain	Shyam et al. (2015)
	Chitosan polymeric micelles functionalized with oleic acid (OA) mannose followed by TAT and MAN-CPP	pVGF	Intranasal	Higher transfection in the brain with intranasal administration	Lamptey et al. (2022)
			Intravenous		
	Chitosan polymeric micelles functionalized with oleic acid (OA) mannose followed by PEN-CPP	pVGF	Intranasal	Higher brain transfection of pVGF in the brain	Gothwal et al. (2023a)
	PEN-conjugated multi-functionalized polymeric micelles	pApoE2	Intranasal	Higher transfection of ApoE2 in the brain	Gothwal et al. (2023b)
Exosomes	CPP RVG on exosome surface protein Lamp2b	BACE1 siRNA	Intravenous	Significant knockdown of BACE1 protein and β amyloid 1-42	Alvarez-Erviti et al. (2011)
	Exosomes releasing miR-29a and miR-29b are generated through biologic functionalization of secretory cells	miRNA	Intrahippocampally	Significant increase in miR-29 amount regulating BACE1 expression and neuronal apoptosis reversing cognitive impairment	Jahangard et al. (2020)
Polymeric NPs	QSH and CGN conjugated PEGylated Poly(2-(N, N-dimethylamino) ethyl methacrylate)	BACE1 siRNA	Intravenous	Suppressed BACE1 expression and decreased Aβ formation	Zheng et al. (2017)
	PEGylated poly (2-(N, N-dimethylamino) ethyl methacrylate) modified with CPP- CGN and Tet 1	BACE1 siRNA	Caudal vein injection	Decreased BACE1 mRNA level and Aβ plaques with significant hippocampal neurogenesis	Wang et al. (2018b)
	Galactose-modified poly(ethylene glycol)-block-poly [(N-(3-methacrylamidopropyl) guanidinium [Gal-PEG-b-P (Gu)]/poly(ethylene glycol)-block-poly [(N-(3-methacrylamidopropyl) guanidinium-co 2,2,3,3-tetrafluoropropyl methacrylate] [PEG-b-P (GuF)] polymer mixture	BACE1 siRNA	Caudal vein injection	Suppressed the expression of BACE1, thereby decreasing amyloid-β formation	Zhou et al. (2020)
	PEGylated poly(2-(N, N-dimethylamino) ethylmethacrylate) (PEG-PDMAEMA) NPs surface modified with CPP- CGN and QSH	BACE1 siRNA	Intravenous	Inhibited Aβ formation was observed, followed by the suppression of BACE1 expression in both mRNA and protein levels	Guo et al. (2019)
	PEGylated dendrigraft poly-L-lysines modified with CPP- RVG29 and D-peptide	BACE1-AS shRNA	Intravenous	Downregulated the expression of BACE1, reduced Aβ plaques, decreased the formation of intracellular tau-fibrils	Liu et al. (2016)
	Polyethylenimine (PEI) NPs modified by PEGylation (PEG)	ROCKII-siRNA	Intracranial injection	Improved cognitive impairment by enhancing Choline acetyltransferase (ChAT) activity in the hippocampus region	Wen et al. (2014)
	CPP-RVG29 modified PEGylated PLGA	BACE1-AS shRNA	Intravenous	Downregulated BACE1 enzyme and amyloid-beta formation	Lv et al. (2020)

## 5 Advancements in nucleic acid delivery for AD therapy

Currently, there are no evidence of clinical studies utilizing NPs for nucleic acid delivery to the brain against AD. However, there are ongoing open-label phase 1 clinical trial using adeno-associated virus serotype 2 (AAV2) for BDNF and APOE2 delivery for AD. ASOs approved for clinical use are efficiently distributed within the brain and spinal cord, highlighting their potential in treating neurodegenerative disorders. The stability of ASO formulations is conferred through chemical modifications (Smith et al., 2006). The antisense oligonucleotide, MAPT_Rx_, has been shown to inhibit the formation of tau protein through endogenous ribonuclease H-1 mediated degradation of MAPT mRNA. In preclinical studies, administration of mRNA-targeting ASOs resulted in a significant reduction of intracellular tau in a mouse model of tauopathy. Furthermore, ASO effectively reduced neuronal and cognitive impairments and significantly inhibited the cell-to-cell spread of oligomerized tau in the study (Takeda et al., 2015). The findings of the study, coupled with the safety profile of MAPT_Rx,_ provided the basis for a phase Ib clinical trial to assess its therapeutic potential in human subjects with AD. The study primarily aimed to evaluate safety, with a secondary focus on the pharmacokinetics of MAPT_Rx_ in cerebrospinal fluid (Mummery et al., 2023). An additional exploratory objective was to analyze the concentration of CSF total-tau protein. Mild or moderate adverse events were documented in 94% of patients treated with MAPT_Rx_ and 75% of those who received the placebo. Notably, there were no reports of serious adverse events among patients treated with MAPTRx. In addition, there was a significant decline in CSF total-tau exceeding 50% from baseline at the 24-week mark after the final dose in cohorts receiving either four doses of 60 mg or two doses of 115 mg of MAPT_Rx_ (Mummery et al., 2023). Based on the positive outcome of the Phase Ib trial, Biogen began a Phase II trial in August 2022 to compare two dosages and two injection schedules to placebo in 735 AD patients with moderate cognitive impairment or mild dementia (Alzforum, 2023).^5^ Participants will be given high or low dosage BIIB080 (IONIS-MAPT_Rx_) every 24 weeks, a high dose every 12 weeks, or a placebo for 72 weeks. The primary outcome is the dosage response in changes in Clinical Dementia Rating Scale Sum of Boxes (CDR-SB) from baseline after 76 weeks. Secondary indicators include Activities of Daily Living for Mild Cognitive Impairment (ADCS-ADL-MCI), Alzheimer’s Disease Assessment Scale–Cognitive Subscale (ADAS-Cog 13), Mini Mental State Examination (MMSE), Integrated Alzheimer’s Disease Rating Scale (iADRS), Alzheimer’s disease composite score (ADCOMS), and adverse events. The experiment is planned to end in 2030 (Trial Registration: NCT05399888) (Biogen, 2022).^6^ Several drug products for AD are already available in the market, with the most recently approved drugs by FDA listed in this article. Additionally, there are still ongoing clinical trials exploring various drug options for AD (Tables 4–6).

**TABLE 4:** Nucleic acids in clinical trials for AD.

Nucleic acid	Vector	Trial start date (end date)	Therapeutic goal	Type of trial (status)	Trial ID	Primary outcome measures	References
BDNF	adeno-associated virus (AAV) vector	7 February 2022	Reduction of neuronal loss and rebuilding synapses in the brain of patients with Cognitive Impairment and AD	Open-label Phase 1 trial	NCT05040217	a. The number of individuals with adverse events related to therapy, evaluated on MRI scan	Tuszynski (2022)
		(1 October 2027)		(Recruiting)		b. Assessment of memory via Ray Auditory Verbal Learning Task	
						c. Assessment of Memory with Benson Complex Figure Draw and Memory	
APOE2	adeno-associated virus (AAV) vector	6 November 2019	Maximum tolerable dose, and conversion of APOE protein variants in the CSF of individuals with APOE4 homozygosity into APOE2-APOE4	Open-label Phase 1 trial	NCT03634007	a. Proportion of participants reporting serious adverse events and treatment-related adverse events, 12 months post-trial initiation	Lexeo (2019)
		(November 2024)		(Active, not recruiting)		b. Proportion of study reporting adverse events associated with treatment at each dosage	

**TABLE 5:** Marketed Drug Products for AD recently approved by FDA.

Drug name (active)	Therapy	Company	Trial commencement	Type of clinical trial conducted for AD (trial ID)	Dosage form	References
Rexulti (Brexpiprazole)	Immunotherapy against Aβ aggregates	Otsuka	10 May 2023	Phase 3, multicenter,12-week, randomized, double-blind, placebo-controlled trial (NCT03548584)	Tablet for oral dose	Lee et al. (2023)
Leqembi (lecanemab-irmb)	Immunotherapy against Aβ aggregates	Biogen	6 July 2023	Phase 3, 18-month, multicenter, double-blind, trial (NCT03887455)	Injection for Intravenous Infusion	Practical Neurology (2023)
Aduhelm (Aducanumab)	Immunotherapy against Aβ aggregates	Biogen	7 June 2021	Two Phase 3, randomized, double-blind, placebo controlled (NCT02484547 and NCT02477800)	Injection for Intravenous Infusion	Budd Haeberlein et al. (2022)

**TABLE 6:** Ongoing clinical trials investigating drugs for AD.

Drugs	Therapeutic purpose	Company	Trial	Clinical trial ID	Status
Donanemab	Anti-amyloid-therapy	Eli Lilly & Co	TRAILBLAZERALZ 2 (Phase 3)	NCT04437511	Active, not recruiting
Donanemab	Anti-amyloid-therapy	Eli Lilly & Co	TRAILBLAZER-ALZ 3 (Phase 3)	NCT05026866	Recruiting
Donanemab/Aducanumab	Anti-amyloid-therapy	Eli Lilly & Co	TRAILBLAZER-ALZ 4 (Phase 3)	NCT05108922	Active, not recruiting
Simufilam	Anti-amyloid-therapy	Cassava Sciences	RETHINK-ALZ (Phase 3)	NCT04994483	Recruiting
Simufilam	Anti-amyloid-therapy	Cassava Sciences	REFOCUS-ALZ (Phase 3)	NCT05026177	Recruiting
CT1812	Anti-amyloid-therapy	Cognition Therapeutics	SHINE (COG0201) (Phase 2)	NCT03507790	Recruiting
CT1812	Anti-amyloid-therapy	Cognition Therapeutics	START (COG0203) (Phase 2)	NCT05531656	Recruiting
BIIB080	Anti-tau DNA/RNA-based therapy	Biogen, IONIS Pharmaceuticals	CELIA	NCT05399888	Recruiting
Bepranemab	Anti-tau DNA/RNA-based therapy	Hoffmann-La Roche	Phase 2	NCT04867616	Active, not recruiting

Reference: Huang et al. (2023).

## 6 Conclusion and future perspectives

This review summarized nucleic acid delivery to the brain using different functionalized nanocarrier platforms to bypass biological barriers and improve target specificity. The existing therapies for AD, including amyloid removal antibodies, provide only symptomatic relief to patients without any interference in halting the disease progression. In such scenario, nucleic acid therapy with pDNA, mRNA, siRNA, miRNA, shRNA, and ASOs holds great promise as a novel therapeutic target. However, standalone delivery of nucleic acid poses significant challenges like susceptibility to degradation during circulation or within the endolysosomal pathway of cells, initiation of immune response, and minimal cellular uptake due to its high negative charge density. Encapsulating these nucleic acids in vectors such as functionalized NPs helps to overcome biological barriers and enhance the targeted nucleic acid delivery to the brain. Developing suitable vectors requires multidisciplinary integration due to several factors such as physiological barriers, vector performance after administration, and undesired interaction with tissue and drugs/genes. The presence of the BBB restricts the transportation of exogenous molecules to the brain; therefore, it must be emphasized in vector development.

Furthermore, gene delivery with NPs holds great promise in targeting associated proteins contributing to AD, which are typically considered non-druggable. This technology also has the potential to extend to associated neurological disorders like Parkinson’s Disease and Amyotrophic Lateral Sclerosis, among others, in a similar fashion to viral vectors. For instance, UniQure Biopharma B.V. used viral vectors for phase 1 and 2 clinical trials (NCT04120493 and NCT05243017) for Huntington’s disease treatment. Similarly, Neurologix Inc. used a viral vector for glutamic acid decarboxylase (GAD) gene delivery in Parkinson’s disease patients (NCT00643890). The complexity in the synthesis of these NPs, their optimization catered to clinical application, and the poorly understood fate of the NPs inside the body present a drawback in the success of this therapy in the future. Studies concerning the undesired genetic effects of nucleic acids, like gene silencing and nonspecific gene expression, warrant further investigation. Typically, preclinical investigations are conducted within relatively constrained experimental conditions and by a limited number of animal models. Hence, research should incorporate diverse statistical, mathematical, and biological models to enhance the likelihood of regulatory approvals for further clinical trials. Researchers must also strive to consider the final dosage form for human use while conducting preclinical investigations, ensuring that formulations have the potential to be translated into clinically applicable therapeutics. The recent advancements highlighted in this review article provide a structured comprehension that can inform the utilization of functionalized NPs in gene therapy for AD. We anticipate that the careful selection of scientifically validated genes, specific promoters, and promoter control mechanisms, coupled with the development of functionalized nanocarriers, will bring gene therapy approaches for AD closer to practical implementation. Additionally, harnessing progress in genomics and precision medicine allows for tailoring NP-based therapies to meet the specific needs of AD patients, considering factors such as their genetic predisposition, disease stage, and other relevant variables. As research in this field advances and nanotechnologies evolve, gene therapy is poised to play an increasingly important role in therapeutics against AD, offering new hope for patients and healthcare providers alike.

## Footnotes

1
www.who.int/news-room/fact-sheets/detail/dementia

2
www.biogen.com/medicines.html

3
www.alz.org/alzheimers-dementia/treatments/medications-for-memory

4
www.eisai.com/news/2024/news202404.html

5
https://www.alzforum.org/therapeutics/biib080/

6
https://clinicaltrials.gov/study/NCT05399888/
